# Editorial: From laboratory insights to clinical strategies in cardiogenic shock

**DOI:** 10.3389/fcvm.2026.1837254

**Published:** 2026-04-10

**Authors:** António Tralhão, Doroteia Silva, Christopher Strong, Matteo Mazzola

**Affiliations:** 1 Intensive Care Department, ECMO Reference Center, Cardiothoracic ICU, ULS São José, Lisbon, Portugal; 2 ⁠Intensive Care Service, ULS Santa Maria, Lisbon, Portugal; 3 Faculty of Medicine, Lisbon Academic Medical Center, Centro Cardiovascular da Universidade de Lisboa, Rede de Investigação em Saúde (CCUL@RISE), Lisbon, Portugal; 4 Intensive Care Medicine Department, ULS Lisboa Ocidental, Lisbon, Portugal; 5 Cardiology Department, ULS Lisboa Ocidental, Lisbon, Portugal; 6 Cardiac, Thoracic and Vascular Department, Azienda Ospedaliero-Universitaria Pisana, Pisa, Italy; 7 Department of Surgical, Medical and Molecular Pathology and Critical Care Medicine, University of Pisa, Pisa, Italy

**Keywords:** beta-blockers, cardiogenic shock, critical care, fluid balance, gastrointestinal bleeding, myocardial infarction

Cardiogenic shock (CS) remains the ultimate *frontier* in Cardiac Intensive Care. Despite clinical progress and a recent positive trial on mechanical circulatory support, the persistence of elevated mortality justifies continued research efforts (1, 2). The ongoing challenge prompted the creation of a dedicated Research Topic entitled “From Laboratory Insights to Clinical Strategies in Cardiogenic Shock”. During the submission period, we received 12 manuscripts. Among the Editorial Team, we ultimately selected 4 manuscripts for publication, each one focusing on a different and relevant aspect of CS management.

Due to a great evolution in coronary revascularization technology, ST elevation myocardial infarction (STEMI) mortality has decreased significantly. Some STEMI patients are nowadays at low-risk for major adverse cardiac events and other traditional complications (3), even if the practice of admitting all STEMI patients to Intensive Care Units (ICU) remains routine (4). That practice overuses ICUs resources and drives up healthcare costs (5) without a clear benefit of mortality (6). There is a major need to identify predictors of critical care needs in this population.

Jnani et al. performed a retrospective cohort study of 758 patients admitted with STEMI to a quaternary Cardiac ICU (CICU) during 5 years, analyzing critical care needs and identifying predictors of their use. Interestingly, they found that despite of the entire STEMI cohort having been admitted to the ICU (per hospital's protocol), only 18.8% were critically ill (that is, in need of mechanical or non-invasive ventilation, titratable vasoactive infusions, sedative infusions or temporary mechanical circulatory support). Moreover, most of these ICU needs arose before CICU admission, implying that the majority of patients who do not require critical care needs prior to or during revascularization, are unlikely to develop ICU needs afterwards.

Finally, they found that multivessel disease had increased burden of critical care needs, whereas successful PCI of culprit lesions mitigated those needs, regardless of the culprit vessel. Factors as chronic kidney disease, a Modified Shock Index on admission ≥0.93 and low ejection fraction were independently associated with critical care needs. These data challenge routine ICU admission for all STEMI patients.

Zhang et al. provided an important and timely contribution by highlighting the dynamic nature of fluid balance in patients with acute myocardial infarction (AMI) complicated by cardiogenic shock (AMI-CS). Rather than relying on static assessments, the Authors applied a Group-Based Trajectory Modeling approach to identify distinct fluid balance patterns and their prognostic implications. Four trajectories emerge: stable negative balance, rapid transition to negative balance, persistent positive balance, and high-level decreasing patterns. The key finding is that trajectories characterized by early or sustained negative balance are associated with improved survival, whereas persistent fluid overload is linked to significantly higher mortality. These results remain consistent across multivariable and sensitivity analyses, reinforcing their robustness. Importantly, this study shifts the clinical paradigm from simple fluid resuscitation toward *fluid stewardship*, emphasizing the need for continuous reassessment of volume status. In the setting of cardiogenic shock—where ventricular dysfunction and elevated filling pressures predominate—excessive fluid administration may exacerbate congestion without improving perfusion (7, 8). The study's main strength lies in its conceptual advance: prognosis is not determined by fluid balance *per se*, but by its trajectory over time. This dynamic perspective supports a more individualized and adaptive approach to hemodynamic management**,** encouraging early de-resuscitation strategies once initial stabilization is achieved to prevent fluid overload and congestion.

Bleeding complications are frequent during ICU admission for CS, especially AMI-CS being linked to poorer outcomes (9). After vascular access-site bleeds, upper gastro-intestinal bleeding (UGIB) is the most frequent source (10).

In a single-center retrospective cohort study comprising 253 patients undergoing primary PCI for AMI-CS, Dong et al. aimed to identify predictors of UGIB. During hospital stay, almost 1 out of 4 patients developed a bleeding event according to defined criteria, despite oral proton pump inhibitor (PPI) prescription. After multivariate adjustment, mortality was higher in the presence of UGIB (63.8% vs. 29.7%, *p* < 0.05). The model showed good discrimination (AUC + 0.768) identifying 3 independent predictors of UGIB: baseline SCAI-CS stage D + E, baseline eGFR < 45 mL/min·1.73 m^2^, and baseline LVEF < 36%. Intensity of antithrombotic therapy did not influence the rate of UGIB. However, the model's sensitivity (87.9%), which minimizes missing a bleeding event, contrasts with a somehow limited specificity (52.3%), which is in part intrinsically related to the low discriminative ability of the predictors for the actual source of bleeding. The creation of highly accurate Scores in Critical Care Cardiology is a difficult task (11). However, and based on the work of Dong et al., the application of their risk score could prove useful to early implementation of intravenous PPI, especially in settings where this intervention is not part of usual care.

Although recent randomized trials have challenged the historical practice of routine beta-blocker therapy for all patients after AMI, current guidelines continue to recommend it in patients with reduced left ventricular ejection fraction (LVEF ≤40%) once clinical stabilization has been achieved, given the demonstrated reduction in mortality and recurrent MI (12–15).

In this context, Gao et al. evaluated whether in-hospital initiation of oral beta-blockers influences long-term outcomes in patients with AMI-CS, using data from the China AMI Registry. The study included 744 eligible patients presenting with cardiogenic shock at admission. Overall, 42.7% of patients received oral beta-blockers during hospitalization. During a 2-year follow-up, all-cause mortality was 41.7%. In county-level hospitals (smaller centers), initiation of beta-blockers was associated with significantly higher mortality (HR 1.79; 95% CI 1.03–3.09; *P* = 0.038). When baseline differences were balanced and adjusted, in-hospital beta-blocker therapy remained associated with increased 2-year mortality (HR 1.59; 95% CI 1.18–2.13; *P* = 0.002).

The authors concluded that initiating oral beta-blockers during hospitalization did not confer a long-term survival benefit in this high-risk AMI-CS population and may be harmful, particularly in less experienced centers. Further randomized controlled trials are needed to clarify the role of beta-blockers in AMI-CS and to better individualize therapy following hemodynamic stabilization.

Taken together, these four studies outline a comprehensive framework that bridges pathophysiological insights and clinical decision-making in cardiogenic shock. The first study highlights the importance of early risk stratification in STEMI patients, showing that many do not require intensive care, thus enabling more efficient resource allocation. The second introduces a dynamic perspective on hemodynamic management through fluid balance trajectories. The third study underscores how systemic complications such as GI bleeding reflect multiorgan dysfunction and are strongly associated with worse outcomes. Finally, the fourth challenges established practices, suggesting that early initiation of beta-blockers in cardiogenic shock may offer no long-term benefit and could even be harmful. Collectively, these findings emphasize that CS management can no longer rely only on uniform protocols but must evolve toward a precision-based, bedside-driven approach.
